# Methicillin-resistant *Staphylococcus aureus* in veterinary professionals in 2017 in the Czech Republic

**DOI:** 10.1186/s12917-019-2223-z

**Published:** 2020-01-06

**Authors:** Katerina Neradova, Vladislav Jakubu, Katarina Pomorska, Helena Zemlickova

**Affiliations:** 1 Department of Clinical Microbiology, Faculty of Medicine and University Hospital, Charles University, Hradec Kralove, Czech Republic; 2 National Reference Laboratory for Antibiotics, National Institute of Public Health, Prague, Czech Republic

**Keywords:** Livestock-associated MRSA, Carriage, Veterinarians, Czech Republic, Livestock, Companion animals

## Abstract

**Background:**

Cases of colonization or infection caused by Methicillin-resistant *Staphylococcus aureus* (MRSA) are frequently reported in people who work with animals, including veterinary personnel. The aim of this study was to determine the prevalence of MRSA colonization among veterinary professionals. A total of 134 nasal swabs from healthy attendees of a veterinary conference held in the Czech Republic were tested for presence of MRSA. The stains were further genotypically and phenotypically characterized.

**Results:**

Nine isolated MRSA strains were characterized with sequence type (ST), *spa* type (t) and Staphylococcal Cassette Chromosome *mec* type. Five different genotypes were described, including ST398-t011-IV (*n* = 5), ST398-t2330-IV (*n* = 1), ST398-t034-V (*n* = 1), ST225-t003-II (*n* = 1) and ST4894-t011-IV (*n* = 1). The carriage of the animal MRSA strain was confirmed in 8 cases, characteristics of one strain corresponded to the possible nosocomial origin. Among animal strains were described three *spa* types (t011, t034, t2330) belonging into one dominating clonal complex *spa*-CC11.

**Conclusion:**

According to our results, the prevalence of nasal carriage of MRSA in veterinary personnel is 6.72%. Although we described an increase compared to the results of previous study (year 2008), the prevalence in the Czech Republic is still remaining lower than reported from neighboring countries. Our results also indicate that healthcare - associated MRSA strains are still not spread among animals.

## Background

*Staphylococcus aureus* (*S. aureus*) is a common bacterium adapted to the human host, persistently colonizing the nasal mucosa of 30%, and transiently present in up to 70% of healthy people [1]. Carriers of Methicillin-resistant *Staphylococcus aureus* (MRSA) are infrequent (0.2%) in people with no previous contact with healthcare [2]. The prevalence is higher among healthcare workers, in Europe it shows geographic dependence, and differs from < 1% in northern countries up to > 40% in Southern and Western Europe [3]. Colonization increases the risk of subsequent infection by fourfold [4]. Under eligible conditions colonizing MRSA strains may cause purulent skin and soft tissue infection or serious pneumonia. Animals, mainly livestock were described as MRSA reservoirs [5]. The livestock-associated strains (LA-MRSA) adapted to animal hosts [6]. After frequent and close contact with an MRSA-positive animal, humans can be colonized by these bacteria, but rarely become infected [7]. Not only farmers and livestock breeders, but also veterinary personnel are at higher risk of acquiring MRSA as shown previously.

The aim of this study was to determine the prevalence of MRSA colonization among veterinary professionals attending a veterinary conference for mixed animal practice and to characterize these strains genotypically and phenotypically (determine their antibiotic resistance profiles).

## Results

Of the 134 attendees who agreed to be tested, 119/134 (88.8%) were veterinarians, 6/134 (4.4%) were pharmacists/researchers and 5/134 (3.7%) were veterinary school students; all confirmed animal contact. Among these healthy volunteers various age groups ranging from 22 to 69 years (median 35.5; mean age 37.6) were represented. With regard to type of practice, 76/134 (57%) worked in small-animal practice, 57/134 (42.3%) in mixed practice, and 1/134 (0.7%) worked with livestock only. The other characteristics are summarized in Table 1.

**Table 1:** Main characteristics of 134 volunteers

Type of practice, *n* (%)					
Small animals	76 (57)				
Livestock	1 (0.7)				
Mixed	57 (42.3)				
Animal contact, *n* (%)	Daily (%)	Weekly (%)	Monthly (%)	Less (%)	Total (%)
Small animals	113 (84.3)	12 (9)	5 (3.7)	22 (16.4)	130 (97)
Cattle	8 (6)	4 (3)	9 (6.7)	19 (14.2)	21 (15.7)
Pigs	1 (0.7)	2 (1.5)	3 (2.2)	16 (11.9)	6 (4.4)
Sheep	4 (3)	5 (3.8)	7 (5.2)	21 (15.7)	16 (12)
Horses	7 (5.3)	9 (6.7)	10 (7.4)	1 (0.8)	26 (19.4)
Mean age, years, range	35.5 (22–69)				
Mean length of clinical practice, years (range)	10 (1–45)				
Male, *n* (%)	46 (34)				
Work position, *n* (%)					
Veterinary professional	119 (88.8)				
Pharmacist, researcher	6 (4.4)				
Student	5 (3.7)				
Country of work					
Czech Republic	119				
Slovakia	8				
Belgium	1				
Hospitalization within past 30 days, *n* (%)					
Yes	10 (7.4)				
No	124 (92.6)				
Healthcare-worker in household, *n* (%)					
Yes	22 (16.4)				
No	112 (83.6)				

*Staphylococcus intermedius/pseudintermedius*, a common pathogen in dogs and cats, was identified in 2/134 swabs (1.5%). *S. aureus* was confirmed in 40/134 samples (29.9%), of which 9/40 (6.72%) were MRSA strains all carrying *mec*A gene. Overall, in this study group there were more women (88/134, 66%) than men (46/134, 34%).

Nine isolated MRSA strains were characterized with sequence type (ST), *spa* type (t) and staphylococcal cassette chromosome *mec* (SCC*mec*). Five genotypes were described, including ST398-t011-IV (*n* = 5), ST398-t2330-IV (*n* = 1), ST398-t034-V (*n* = 1), ST225-t003-II (*n* = 1) and ST4894-t011-IV (*n* = 1). To determine the clonal relatedness of the isolates, Based Upon Repeat Pattern (BURP) analysis was performed according to the assigned *spa* types. S*pa* type t011 was determined as a founder of the cluster *spa*-CC11. S*pa* t2330 and t034 belonged to the same aforementioned cluster, while the isolate with *spa* type t003 was singleton. In accordance with their *spa* and SCC*mec* type, the strains showed a typical antibiotic resistance profile. Except for the one *spa* type t003, the isolates were resistant to tetracycline, and strains t011, t2330 were additionally resistant to gentamicin and ciprofloxacin (Table 2).

**Table 2:** MRSA strains characteristics

MRSA strain	V13	V17	V26	V27	V45	V73^c^	V78	V122	V129
*Spa* type	t011	t003	t2330	t011	t011	t034	t011	t011	t011
Repeat succession	08–16–02-25-34-24-25	26–17–20-17-12-17-17-16	08–16–02-25-34-24-25-25	08–16–02-25-34-24-25	08–16–02-25-34-24-25	08–16–02-25-02-25-34-24-25	08–16–02-25-34-24-25	08–16–02-25-34-24-25	08–16–02-25-34-24-25
*Spa* clonal complex	CC11	singleton	CC11	CC11	CC11	CC11	CC11	CC11	CC11
MLST type	ST398	ST225	ST398	ST398	ST398	ST398	ST4894	ST398	ST398
Allelic profile ^a^	3–35–19-2-20-26-39	1–4–1-4-12-25-10	3–35–19-2-20-26-39	3–35–19-2-20-26-39	3–35–19-2-20-26-39	3–35–19-2-20-26-39	3–35–1-2-20-26-39	3–35–19-2-20-26-39	3–35–19-2-20-26-39
SCC *mec* type	IV	II	IV	IV	IV	V	IV	IV	IV
Antibiotic resistance profile ^b^	CXT, TET, GEN, CIP	CXT, ERY, CLI, CIP	CXT, TET, GEN, CIP	CXT, TET, GEN, CIP	CXT, TET, GEN, CIP	CXT, ERY, CLI, TET	CXT, TET, GEN, CIP	CXT, TET, GEN, CIP	CXT, TET, GEN, CIP
Other risk factor	None	None	Hospitalization (3 months ago)	None	healthcare worker contact (daily)	None	None	MRSA positive animal contact (1 week ago)	healthcare worker contact (daily)

^a^ internal fragments of seven house-keeping genes: *arc* (Carbamate kinase), *aro* (Shikimate dehydrogenase), *glp* (Glycerol kinase), *gmk* (Guanylate kinase), *pta* (Phosphate acetyltransferase), *tpi* (Triosephosphate isomerase), *yqi* (Acetyle coenzyme A acetyltransferase)

^b^ *CXT* cefoxitin, *TET* tetracycline, *GEN* gentamicin, *CIP* ciprofloxacin, *ERY* erythromycin, *CLI* clindamycin

^c^ Place of work: Slovakia

The MRSA carriers reported contact with large farm animals (pigs, cattle, horses, sheep) only in 3/9 cases, the frequent contact stated one of them, in the remaining 2 cases the contact was less frequent. Interestingly, all of them reported regular frequent contact with small animals (daily 8/9, weekly 1/9). The statistical analysis (Fisher test) did not show a significant association between the carriage of MRSA and the frequent contact with small animals.

## Discussion

MRSA is an important pathogen not only for humans, but also for small animals or livestock and colonization itself brings the risk of future infection. The higher prevalence of MRSA carriage in veterinary personnel has been proven by multiple studies all over the world. The rates in Europe vary from 0.7–19.2% [8, 9]. Traditionally, high prevalence data come from countries with well-developed livestock production, such as Netherlands, Denmark or Germany [10–12]. Type of veterinary practice, frequency of contact with animals, time since exposure and the study design itself are factors leading to international differences in prevalence rates. There is a lack of data describing the situation in the Czech Republic. A study of similar design performed in the Czech Republic in 2008 revealed 0.7% (2/280) colonization of veterinary personnel attending the conference [8], but the strains were rather not animal-related and the rate corresponded with expected community colonization rates [13]. According to our results, the prevalence of nasal carriage of MRSA in veterinary personnel in our country has increased to 6.72% [9/134, 95% exact Confidence Interval (CI) (3.12, 12.37)].

The most prevalent livestock-associated MLST type in Europe is ST398, we confirmed dominance of this clone also in the Czech Republic. Animal related MRSA strains belonged to *spa* t011, t034 and t2330. The first two are ranked as the most common *spa* types in European conditions together with t108 and t567 [14]. Moreover, an isolate *spa* t034 was confirmed in a veterinarian from neighboring country (Slovakia), who was in contact with both companion animals and livestock. *Spa* t034 had high prevalence among pigs originated from Slovakia [15]. All animal strains showed typical resistance to tetracycline, an antibiotic frequently used in food animal production [16].

The strain ST225-t003-II was a singleton and differed from others in antibiotic susceptibility profile. It had been previously described as predominant in hospitals in the Czech Republic, central Germany, and western Poland [17]. Carrier of this strain did not confirm any contact with the healthcare facility or residence with a healthcare worker in the 30-day period before screening, however, we could not rule out the possibility of contact before the 30-day period questioned. We could not exclude that the strain could possibly have originated from companion animals [18].

MRSA carriage or infections were described mostly in farm animals, such as pigs [19], cattle [20], sheep [21], horses [22] or poultry [23]. Transmission to humans has been documented, mostly after prolonged, repeated and close contact with a colonized animal [24] or through a contaminated vehicle such as meat [25] or dairy products [26]. Several studies confirmed the presence of MRSA ST398 in pigs, goats, cow and sheep and their meat and milk products in the Czech Republic [27–30]. The strains isolated from pigs and pork meat belonged to ST398, whereas the strains from cattle were multiple ST types with the *spa* type t011 and t034 detected most frequently [31].

All colonized veterinarians stated frequent contact with small animals, and regular contact with livestock concerned only three of them. Therefore the sporadic finding of *S. intermedius/ pseudintermedius* in our test group is surprising. Their sharing between pets and humans has been well documented [32]. *S. aureus* is not a typical commensal bacterium in companion animals, being described in less than 10% and even lower (0.7%) in the case of MRSA carriage [33, 34]. MRSA isolates circulating in dogs and cats belong mostly to nosocomial clones often identified in humans [18]. This indicates that MRSA isolated in companion animals may originate in humans and those animals are a reservoir for possible human re-infection [35]. We observed, MRSA carriage in veterinary personnel was primarily associated with contact with small animals. Its statistical significance has not been confirmed, the frequency of positive finding is too low to be generalized.

## Conclusion

This study showed increasing prevalence of MRSA carriage in veterinary personnel with confirmed presence of both animal and nosocomial strains. Attention should be given to the increased colonization rates in this occupational group, especially on admission to hospitalization. Occupational history should be supplemented in clinical practice with a statement about contact with animals. This risk factor should not be underestimated before surgical procedures because of the undeniably higher occurrence of infectious complications in these individuals and MRSA screening should be provided.

## Material and methods

### Study population

We addressed attendees of the veterinary conference VETclasses 2017, held in Hradec Kralove, Czech Republic, 23. - 24. 9. 2017. The target groups were both small-animal and livestock practitioners. Among 436 participants, there were 334 practicing veterinarians, and 102 nurses, technicians and other personnel involved in industry or research. Most of them were from the Czech Republic, but a few representatives from Slovakia and Belgium were also represented. Total of 134 volunteers agreed to be screened, which is approximately 3% of the practicing veterinarians in Czech Republic. According to the Chamber of Veterinary Surgeons of the Czech Republic, there were 4205 private veterinarians registered, which forms the majority of practicing professionals in the Czech Republic (accessed 26 August 2019). In our country small animal veterinary practitioners make up 70%, the rest are involved in mixed animal practice, livestock specialized veterinarians have minimal representation. The Ethics Committee of the Faculty Hospital in Hradec Kralove gave permission to carry out the study on human volunteers.

### Sample collection

Bilateral nasal swab specimens (~ 1 cm into each nostril) were collected with sterile cotton-tipped swabs, stored in transport medium (Copan Transystem®) and transported immediately for laboratory processing. The sample collection was voluntary and anonymous, and additional data were obtained: demographic data, data on exposure to animals or a hospital environment, place of work, job description, type of clinical practice (small animals, mostly dogs, cats or large animals, horses, pigs, ruminants), known exposure to an MRSA positive-animal, previous hospitalization within 30 days, residence with a healthcare worker. The questionnaire was developed for the purposes of this study (Additional file 1).

### Bacterial strains

Nasal swabs were cultured for 18 h on Sheep Blood Agar (Oxoid™ Columbia Blood Agar Base, Thermo Scientific™) and chromogenic agar MRSA Select™ (Bio-Rad). *S. aureus* was identified morphologically, identification was confirmed by Matrix-Assisted Laser Desorption/Ionization Time of Flight Mass Spectrometry (MALDI-TOF MS, Bruker Microflex LT™, Bruker Daltonics). MRSA was detected by cefoxitin resistance [36] and confirmed by detection of *mec*A and *mec*C genes by PCR [37].

### Antibiotic susceptibility testing

Testing and evaluation of Minimal Inhibitory Concentrations (MIC) in MRSA strains was performed by broth microdilution method according to standard ISO 20776-1 [38]. Susceptibility to erythromycin, clindamycin, linezolid, chloramphenicol, tetracycline, ciprofloxacin, trimethoprim/sulfamethoxazole, gentamicin and vancomycin was tested.

### *Spa* typing and Based upon repeat pattern (BURP) analysis

The typing was performed with primers *spa*-1113f (5’TAA AGA CGA TCC TTC GGT C − 3′) and *spa*-1514r (5′-CAG CAG TAG TGC CGT TTG CTT -3′) [39]. The software Ridom StaphType™ (ver. 2.2.1; Ridom GmbH) was used for sequence and BURP analysis. Resulting clonal clusters (*spa*-CCs) were composed of ≥2 related *spa* types and clustered only if their cost value was ≤4 and had at least 5 repeats [40]. The algorithm counts with repeat duplication, deletion and point mutation when assessing if different *spa* types are related.

Multilocus Sequence Typing (MLST) was performed as described previously [41]; the allele types and the resulting STs were assigned using software BioNumerics (ver.7.0; Applied Maths).

### SCC*mec* typing

The SCC*mec* types were identified using multiplex PCR based on identification of specific genes within J regions of particular cassettes (I to V) as described previously [42].

### Statistical analysis

The statistical analyses (Confidence Interval, Fisher test) was performed using NCSS 11 Statistical Software (2016). NCSS, LLC. Kaysville, Utah, USA, ncss.com/software/ncss.

## Supplementary information

**Additional file 1:** Questionnaire. Questionnaire used to obtain additional data from volunteers in this study.

## Data Availability

All data generated or analyzed during this study are included in this published article.

## Abbreviations

*arc*: Carbamate kinase
*aro*: Shikimate dehydrogenase
BURP: Based upon repeat pattern
CC: Clonal complex
CI: Confidence interval
CIP: Ciprofloxacin
CLI: Clindamycin
CXT: Cefoxitin
ERY: Erythromycin
GEN: Gentamicin
*glp*: Glycerol kinase
*gmk*: Guanylate kinase
LA-MRSA: Livestock-associated methicillin-resistant *Staphylococcus aureus*
MALDI-TOF MS: Matrix-assisted laser desorption/ionization time of flight mass spectrometry
MIC: Minimal inhibitory concentrations
MLST: Multilocus sequence typing
MRSA: Methicillin-resistant *Staphylococcus aureus*
PCR: Polymerase chain reaction
*pta*: Phosphate acetyltransferase
*S. aureus*: *Staphylococcus aureus*
SCC*mec*: Staphylococcal Cassette Chromosome *mec*
*spa*-CCs: *spa* clonal clusters
ST: Sequence type
t: *spa* type
TET: Tetracycline
*tpi*: Triosephosphate isomerase
*yqi*: Acetyle coenzyme A acetyltransferase
