# Supplementary material for: Methicillin-resistant Staphylococcus aureus in veterinary professionals in 2017 in the Czech Republic
Source: BMC Vet Res. 2020 Jan 6;16:4. doi: 10.1186/s12917-019-2223-z (PMC6945690; doi:10.1186/s12917-019-2223-z)
Supplement: Supplementary file 1 — Additional file 1: Questionnaire. Questionnaire used to obtain additional data from volunteers in this study. [file 12917_2019_2223_MOESM1_ESM.doc]

**Questionnaire**

**Methicillin-resistant *Staphylococcus aureus* in veterinary professionals in 2017 in the Czech Republic**

VETclasses 2017 Hradec Kralove, Czech Republic, 23. - 24. 9. 2017

(mark your answers with a cross)

sample number:

(to be completed by the laboratory)

Age:

Sex:

Region of employment:

Type of Clinical Practice: Small animals

|  |
| --- |
|  |

Livestock

Pigs

|  |
| --- |
|  |
|  |
|  |

Horses

Cattle

Sheep

Frequency of contact with animals:

(with regard to the last 6 months of praxis)

|  | Pigs | Horses | Cattle | Sheep |
| --- | --- | --- | --- | --- |
| Daily |  |  |  |  |
| Weekly |  |  |  |  |
| Monthly |  |  |  |  |
| Less frequently |  |  |  |  |

Hospital stay during the last 30 days: YES NO

Shared household with healthcare worker: YES NO
